# Ag flake/silicone rubber composite with high stability and stretching speed insensitive resistance via conductive bridge formation

**DOI:** 10.1038/s41598-020-61752-2

**Published:** 2020-03-19

**Authors:** In Seon Yoon, Sun Hong Kim, Youngsu Oh, Byeong-Kwon Ju, Jae-Min Hong

**Affiliations:** 1 Display and Nanosystem Laboratory, Department of Electrical Engineering, Korea University, Seoul, 02841 Republic of Korea; 2 Department of Electrical and Computer Engineering, Inter-University Semiconductor Research Centre, Seoul National University, Seoul, 08826 Republic of Korea; 3 Photo-Electronic Hybrids Research Centre, Korea Institute of Science and Technology (KIST), Seoul, 02792 Republic of Korea; 4 Institute of Advanced Composite Materials, Korea Institute of Science and Technology, Jeonbuk, 55324 Republic of Korea

**Keywords:** Materials for devices, Soft materials

## Abstract

High stability, stretchable speed insensitive properties, high stretchability, and electrical conductivity are key characteristics for the realisation of wearable devices. However, conventional research is mainly focused on achieving only high stretchability and electrical conductivity. Studies on the stability and stretching speed insensitive properties generally require complex fabrication processes, which are in need of further improvement. In this study, we propose a facile formation of a conductive bridge in composites by using surface damage and the viscoelastic property of the polymer. Surface cracks due to repeated stretching cycles formed conductive bridges via stress relaxation of the viscoelastic polymer matrix. The conductive bridge resulted in the conductor having highly stable resistance values at target strains and stretching speed insensitive resistance, even at stretching speeds that were 20 times faster than the minimum.

## Introduction

There have been great interests in stretchable conductors for the past few decades; as such, many methods for the fabrication of highly stretchable and conductive conductors have been researched. Instead of conventional rigid substrates and electrodes, stretchable substrates and conductors have been studied via various methods such as printing with composite ink^1–4^, prestraining^5–8^, and embedding nanowires in a stretchable matrix^9–12^. In addition, practical applications such as stretchable displays^13–17^, wearable motion sensors^18–21^, and stretchable energy storage devices^22–25^ have been studied using the stretchable conductors mentioned above. Furthermore, stretchable conductors are studied as an interconnection electrode for future transparent devices, such as flexible photodetectors^26,27^, and tiny devices, such as memristors and memory^28,29^. These researchers have made great progress in making highly stretchable and conductive conductors. However, for practical uses of these stretchable devices, there are new stability conditions, where the conductors are required to withstand thousands of stretching/contraction cycles and be independent of the stretching speed.

From this point of view, self-healing polymer conductors are gaining more interest as a promising method to modify the stability of conductors^30–32^. Cracks are generated by repeated stretching/contraction cycles; this can make the conductive pathway longer, or even break it, making the conductor dielectric. Even after the conductors are separated, a self-healing polymer conductors can reform the network and make it conductive again. Although this healing process restores mechanical and electrical properties, there are still problems to be solved. Self-healing polymer conductors require many conditions such as broken conductors should be placed to contact each other and number of restores are limited. So, forming conductive bridge along the broken conductors can be an alternative for highly stable and stretching speed insensitive conductor.

Recently, the concept of conductive bridges in stretchable conductors was first introduced by Lee^33^. Carbon black conductors in conducting composites were damaged due to stretching, resulting in a rapid increase of electrical resistance. Lee found that adding carbon nanotubes increased the electrical conductivity via the formation of conductive bridges between separated carbon black clusters. Thus, the formation of conductive bridges is advantageous in terms of the ease of fabrication and ease of preserving the conductive path in comparison with self-healing polymers. However, position and direction of the carbon nanotubes could not be changed arbitrary, also the composite showed poor electrical property. Therefore, the concept of conductive bridge needed further improvements in terms of forming process and consisting materials.

In this paper, we suggest new method to fabricate stretchable conductors with high stability and strain-insensitive resistance due to the formation of conductive bridges induced by surface damage. Cracks are usually fatal defects for stretchable conductors. However, in the case of conductors with a viscoelastic polymer matrix, cracks can work as driving force for the formation of conductive bridges. At the crack, the composite curls up and down due to its viscoelastic property, and the closely packed conductive filler network forms a conductive bridge. In our study, this conductive bridge resulted in high stability for the conductor, with stable electrical resistance for 1000 cycles and stretching speed insensitive electrical resistance.

## Results and discussion

**Figure 1:**
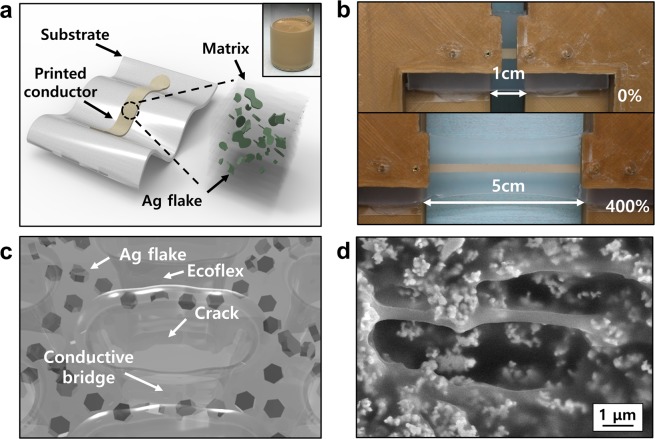
(**a**) Schematic of a stretchable Ag flake/silicone rubber conductor on a silicone substrate. The cube illustrates the microstructure of the conductor. The inset shows the conductive ink before printing. (**b**) Photographs of the stretchable conductor at relaxed (0% strain) and stretched (400% strain) states. (**c**) Schematic illustration of a conductive bridge and microstructure in the conductor. (**d**) Scanning electron microscope (SEM) image (top view) of the stretchable conductor at a generated crack.

Figure 1 shows schematics and samples of the stretchable conductor, composed of silicone rubber (Ecoflex) and Ag flakes, and the cracks induced by multiple elongation and concept of the conductive bridge. The inset of Fig. 1a shows a conductive ink comprising a conductive filler (Ag flakes), silicone rubber, and a solvent (4-methyl-2-pentanone), which is used to enable the uniform dispersion of Ag flakes in the silicone rubber matrix. The conductor showed a high stretchability of 400–500%, even after sintering at 130 °C, as shown in Fig. 1b. The same silicone rubber material for both the matrix and substrate to eliminate the modulus mismatch^34,35^.

The prepared samples went through a number of stretching/contraction cycles, creating cracks in the stretchable conductors as shown in Fig. 1c,d. The Ag flakes at the cracks curl up and down, forming a closely-packed conductive network i.e., a “conductive bridge.” Due to the viscoelastic property of the stretchable matrix, the cracks efficiently relaxed the stress induced to the conductors which results in stable structure of the conductor^36^.

Usually, well-dispersed structure is desired in metal powder/polymer composites for mechanical stability. It is well known that the aggregation of filler particles results in crack generation and propagation which is not good for mechanical strength. However, we wanted to induce cracks inside of the conducting composites to enhance electrical properties.

We induced cracks by fabricating a conductor with non-uniform dispersion of the filler. Incorporation of a uniformly dispersed filler in the rubber matrix improves the cyclic durability^1,37^. However, if the fillers are partially aggregated, the parts without the fillers tend to generate cracks. The simulation results demonstrate that the aggregation of fillers causes stress concentration at the parts without fillers (Fig. S1). The conductor with uniform dispersion exhibited well-distributed stress along the whole conductor, as illustrated in Fig. S1a,b. In contrast, the conductor with non-uniform dispersion exhibited concentrated stress under stretching (Fig. S1c,d). The stress was concentrated at the filler-free areas, which results in a crack generation.

To fabricate the conductor with non-uniform dispersion, which forms a conductive bridge through crack generation, we deliberately used an insufficient amount of solvent. A solvent works as a dispersion agent of conductive filler at conductive ink. Therefore, using a sufficient amount of solvent in the conductive ink is essential for the uniform dispersion of the conductor. However, we deliberately used an insufficient amount of solvent, which resulted in the non-uniform dispersion and Ag flake clusters of the fabricated conductor. Figure S2 depicts the crack generations of the conductor with uniform and non-uniform dispersion. As illustrated in Fig. S1a, no cracks were observed on the stretched surface of the conductor with uniform dispersion after 5 cycles. In contrast, conductor with non-uniform dispersion exhibited a relatively vulnerable property to the cyclic test, and crack generations were observed on the surface after 5 cycles (Fig. S2b).

**Figure 2:**
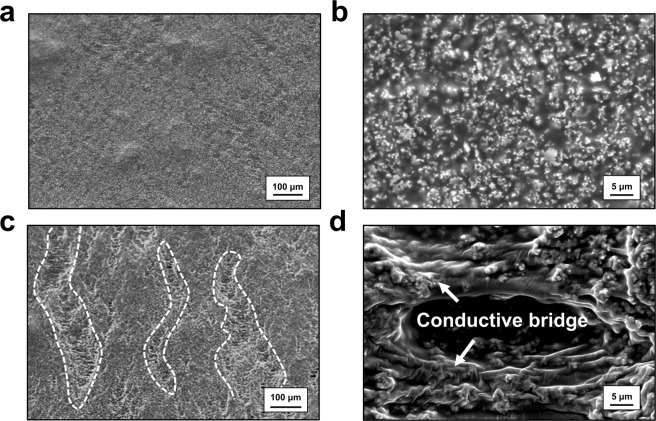
SEM images of stretched conductors at 100% strain. Initially stretched (**a**) large-area image and (**b**) magnified image. (**c**) Large area image and (**d**) magnified image of stretched conductor after 1000 cycles of stretching/contraction.

For further investigation of the formation of the conductive bridge, we observed the surface state of the conductors before and after the cycle test. In Fig. 2, the surface SEM images of stretchable conductors for samples with and without a conductive bridge are depicted. Figure 2a,c show large-area SEM images of 100% stretched samples before and after 1000 stretching/contraction cycles. In Fig. 2a, only few wavy elements are seen, without obvious cracks. In contrast, after 1000 stretching/contraction cycles, the conductor clusters were separated, with white string-like conductive bridges (Fig. 2c). The white dashed lines indicate valleys of separated conductors, meaning huge cracks are generated. Figure 2b,d show the magnified images of the stretchable conductors. In the first stretching, neither cracks nor conductive bridges are observed; only well dispersed Ag flakes can be seen. After repeated cycles, we can easily find a large number of cracks alongside conductive bridges. These conductive bridges connect conductor clusters, preventing the complete loss of electrical conductivity. In addition, we can confirm that a number of Ag flakes are embedded in the bridges between conductor clusters, resulting in the formation of a conductive pathway along the string-like bridges.

**Figure 3:**
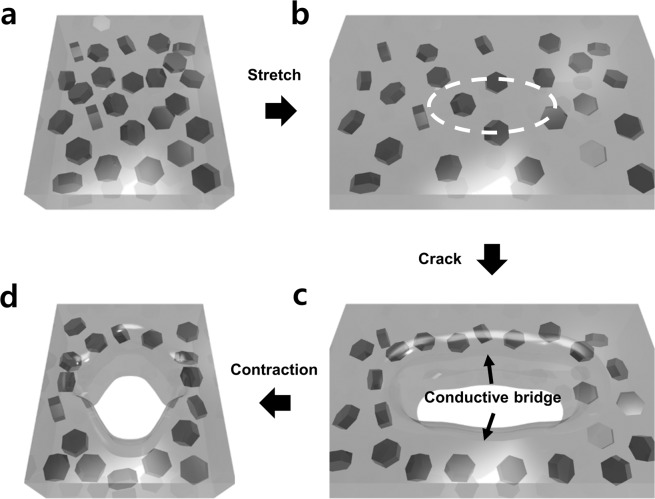
Schematic illustration of conductive bridge formation by crack generation. (**a**) Normal state; (**b**) stretched state; (**c**) crack generation; (**d**) crack disappearance step.

**Figure 4:**
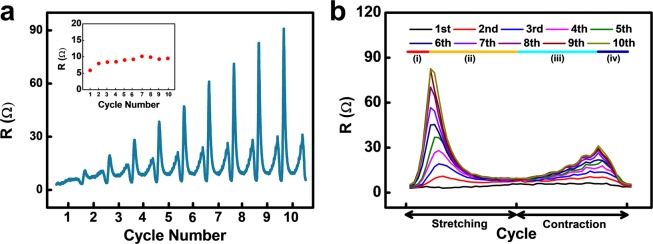
(**a**) Resistance-peak-shape variation of the conductor for 10 cycles (100% strain). Inset shows separately collected resistance at target strain (100% strain). (**b**) Overplot of the resistance tendency for 10 cycles.

Based on the SEM images, we suggested a conductive bridge mechanism by 3D modelling, as shown in Fig. 3. At the beginning, Ag flakes are dispersed in silicone rubber and the interparticle distance is sufficiently small, which results in low resistance (Fig. 3a). As the conductor is stretched, the interparticle distance between conductive particles gets larger, resulting in the increase of resistance. This variation is shown in Fig. 3a,b. During the first cycles of stretching/contraction, there are no cracks. However, as more stretching/contraction cycles are conducted, cracks are generated. The composite at the cracks curls up and down and relaxes due to its viscoelastic property (Fig. 3b,c). Before stretching, the inter-bondings of the polymer are randomly oriented, thereby linking the polymers. Due to stress, these bondings are rearranged along the strain direction, gradually relaxing the stress^38^. At this point, the Ag flakes can move within the polymer and this relaxation results in the concentration of conductive particles along the sides of the crack, forming conductive bridges^39,40^. As the conductive bridge is formed, the electrical resistance decreases (curve (ii) in Fig. 4b). During the contraction step, the conductor shortened and cracks are filled by the surrounding composite, as shown in Fig. 3d. However, this contraction also randomises the conductive filler relaxation, which results in a small amount of increase in resistance, represented by curve (iii). At last, the cracks are almost filled, and the conductor is contracted; the electrical resistance starts to decrease (curve (iv) in Fig. 4b), and the original shape is restored (Fig. 3a).

To investigate the effect of the conductive bridge on electrical resistance, we analysed the results of 10 cycles of stretching/contraction at 100% strain, which is the typical strain for normal skins and joints^41^. The momentary increase in resistance at the beginning has been reported as overshooting^36,42^. However, the reason behind the obvious peak shape change and increase in resistance during the contraction step has not been reported. At the beginning of the cycle test, the peak demonstrated a normal sharp shape; the peculiar-shaped peaks appeared when the test was continued (Fig. 4a). To analyse the change in the peak shape, we overplotted the data of 10 cycles, as depicted in Fig. 4b. As the number of cycles progressed from 1 to 10, the peak shape changed, from a normal sharp peak to a hump-shaped peak, with two peaks on each side of the cycle. We divided the hump-shaped peak into 4 parts, from (i) to (iv), for further analysis. This change implies that cracks occurred in the conductor due to poor durability. An encouraging point of this unique phenomenon is that, although a rapid resistance change was observed, the conductor exhibited a stable resistance value at the maximum strain of the stretching/contraction process (100% strain).

**Figure 5:**
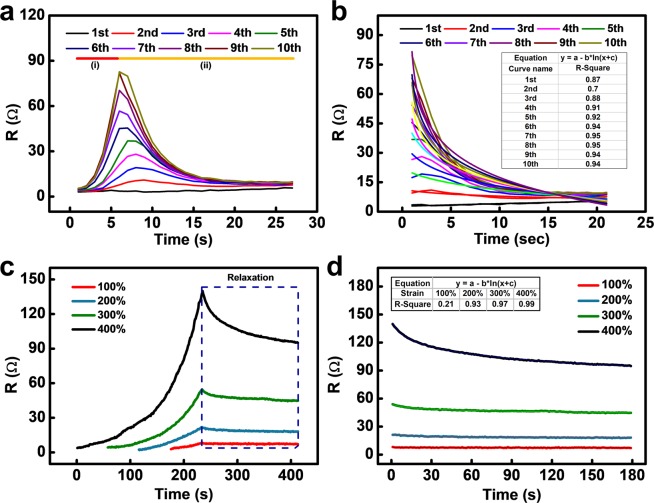
(**a**) Overlapped resistance variation during stretching for 10 cycles. (**b**) Magnified resistance relaxation and fitted line corresponding to curve (ii). (**c**) Resistance variation for stretching and holding test. (**d**) Magnified resistance variation and fitted line corresponding to relaxation curve.

For further analysis of the hump-shaped resistance peak, we focused on the stretching peak for 10 stretching cycles (Fig. 5a,b). As depicted in Fig. 5a, the stretching part of the resistance graphs is divided into two parts as the number of cycles increases. In the middle of the stretching process, the resistance suddenly starts to decrease (curve (ii) in Fig. 4b); this turning point occurs earlier for the later cycles, until saturation at the 5^th^ or 6^th^ cycle. This decrease in resistance corresponds to Figs. 3b,c, which indicates the formation of the conductive bridge. We focused on the unusual decreasing curve under stretching and fitted it with a logarithmic curve, i.e., the well-known viscoelastic relaxation curve^36^.

For the first cycles, the resistance curve was not in agreement with the logarithmic fit due to the relatively low relaxation and high noise values. However, for subsequent cycles, the R-square value increased to about 0.94, which implies that the results were in good agreement with the equation. This equation is based on the viscoelastic relaxation, which implies that the hump-shape peaks are related to the viscoelastic property of the stretchable conductor matrix. Considering the fact that the hump-shape peaks occurred after repeated cycles and that the resistance decreased during stretching, it is evident that these phenomena are related to crack generation and viscoelastic relaxation of the crack. Therefore, we studied the relaxation phenomenon due to the viscoelastic property without crack generation. The relaxation of the electrical resistance due to the viscoelastic polymer matrix was measured under various strains. In Fig. 5c, the resistance graph demonstrates a relaxation of 180 s after stretching at 100, 200, 300, and 400% strain. Under stretching, the electrical resistance increases, which is represented by the exponential curves. However, just after the stretching process is completed, the resistance starts to decrease. As more strain is applied to the conductors, the relaxation gap becomes larger and is proportional to the degree of strain. The resistance decreased by 32.2% from the maximum resistance at 400% strain; in contrast, only a 13.3% decrease was observed at 100% strain. The resistance graph and fits with logarithmic curves when the samples are held after stretching are depicted in Fig. 5d. For higher strain values, i.e., over 200%, the fit had a high R-square value, which implies that the curve is in accordance with the viscoelastic relaxation model. In the case of 100% strain, the low R-square value seems to be a result of the noise owing to the low resistance variation. It has been shown that this resistance relaxation is caused by the stress relaxation of the substrate; hence, we also measured the stress relaxation of the silicone rubber substrate^36^. To compare the actual stress relaxation of the substrate and resistance relaxation, both relaxations were measured at 300% strain, as illustrated in Supplementary Fig. S3. The stress of the stretched substrate after relaxation was 5.4% lower than the maximum stress; however, the electrical resistance dropped by 17.1% compared to the maximum resistance. This relaxation gap implies that the resistance relaxation is affected by not only the substrate relaxation but also the internal relaxation of the stretchable matrix of the conductors.

**Figure 6:**
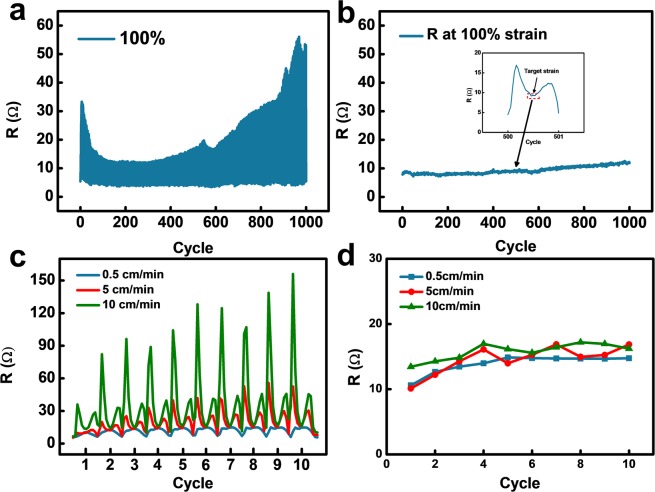
(**a**) Resistance variation of the conductor subjected to cyclic testing at 100% strain. (**b**) Separately collected resistance at target strain (100%) for 1000 cycles. (**c**) Resistance variation of the conductor subjected to cyclic testing for three different stretching speeds (100% strain). (**d**) Separately collected resistance at target strain (100%) for 10 cycles.

Due to the viscoelastic properties of the polymer matrix, the conductive bridge has several advantages; it is highly stable and renders the electrical resistance insensitive to the stretching speed. To investigate the advantages of conductive bridges for a stretchable conductor, the resistance variations for 1000 cycles and different stretching speeds were measured; the *in-situ* measurement results are presented in Fig. 6. At the first glance, in Fig. 6a, the resistance peak seems to be irregular and unpredictable, which may indicate the low durability of the conductors. However, this irregularity results from initial sharp peaks induced by cracks. Interestingly, the resistance variations at the target strain (100% strain) are relatively stable. Figure 6b depicts separately collected resistance values at the target strain during the stretching/contraction process for 1000 cycles. At the beginning, a slight fluctuation of resistance is observed, which seems to be related to crack formation and residual elongation of the polymer substrate. After this fluctuation, the resistance demonstrated a high stability for the remaining 1000 cycles, especially when compared to previous studies^3^.

In the case of high stretching speeds, viscoelastic polymers move rapidly to relax external stress. In contrast, at low stretching speeds, a polymer matrix moves slowly. Hence, a stretchable conductor with a viscoelastic polymer will be insensitive to the stretching speed, which is well expressed in Fig. 6c,d. In Fig. 6c,d, the resistance variation for 10 cycles of stretching/contraction for three different stretching speeds is demonstrated. We increased the stretching speed by almost 20 times the minimum speed, from 0.5 cm/min to 10 cm/min. The initial peaks, which are related to crack generation, increased sharply with higher stretching speeds; however, the relaxing speed also increased, which resulted in stretching speed insensitive electrical resistance at 50% strain. For further investigation of the relation between the stretching speed and the resistance at the target strain, separately collected resistance variations at the target strain for 10 cycles are shown in Fig. 6d. At the target strain (100% strain), the stretchable conductor showed stretching speed insensitive resistance, even for speeds that were 20 times higher (10 cm/min). In conclusion, the resistance slightly increased after 10 cycles, but was independent of the stretching speed and the number of cycles. Considering that previously researched stretchable conductors showed increasing resistance variation gaps with higher stretching speeds, the stretching speed insensitive resistance found for our samples is a superior characteristic for stretchable electronics^43,44^.

In summary, we demonstrated the fabrication of a stretchable conductor with high stability and stretching speed insensitive resistance due to the formation of a conductive bridge using the viscoelastic property of the stretchable matrix. Repeated stretching/contraction cycles of the conductor with non-uniform dispersion of the filler resulted in damage to the conductor surface, i.e., the formation of cracks, which formed conductive bridges. Due to these conductive bridges, the conductor maintained high stability under repeated stretching/contraction cycles and had a similar resistance value at target strains under different stretching speeds. This paradoxical use of surface damage as a conductive bridge represents an important step towards the realisation of highly stable and stretching speed insensitive wearable devices.

## Methods

### Preparation of stretchable composite conductors

We fabricated stretchable composite conductors having relatively low durability for stretching/contraction cycles. The conductors were prepared in three steps: (i) fabrication of the composite inks, (ii) printing, and (iii) heat treatment. The composite inks comprised a conductive filler (Ag flakes, Daejoo Electronics, DSF-500MWZ-S), silicone rubber (Ecoflex 00–30, 1:1 mass ratio of the base to the curing agent, Smooth-on), and a solvent (4-methyl-2-pentanone, Daejung Chemicals). The mass ratio of the three components was 17:4:3. The mass ratio of 17:4:6 was used to fabricate conductors with uniform dispersion. The inks were stirred for 1 h and printed on a silicone rubber substrate with a metal mask. The printed ink was thermally cured using a programmable oven (OF-02PW, JEIO TECH) in the following steps: 60 °C for 1 h for partial curing, 110 °C for 2 h for the evaporation of the solvent, and 130 °C for 2 h for the sintering of Ag flakes and full curing of the silicone rubber.

### Stretching tests

Stretching/contraction tests were carried out using a custom-made stretching system (shown in Supplementary Fig. S4). The resistance probes and motor fixtures were designed using Rhinoceros (Robert McNeel & Associates) and fabricated using a 3D printer (Anartz Engine). A conventional stepper motor was operated using an Arduino Uno microcontroller, and a stretching program based on the C programming language was designed. The variables for the strain tests were the number of cycles, the length of a cycle, and the motor speed. To make the stretching rate constant, increasing force was applied to the conductor. Figure S5 depicts the applied force up to 300% strain. Test-probe pins with a spherical tip (P75-D), a NI USB-4065 digital multimeter, and a LabVIEW (National Instruments) program were used to measure the electrical resistance. The stretching speeds tested in this study were 0.5, 5, and 10 cm/min.

### Characterisation

An AI USB-4065 digital multimeter was used to measure the changes in the resistance during stretching. Surface SEM images of conductors were obtained using a field-emission scanning electron microscope (FE-SEM, Inspect F50 and E-SEM, FEI). Stress-strain curves and stress-time curves were obtained using a universal testing machine (Korea Polymer Testing & Research Institute). The stretching speeds were 0.5, 5, and 10 cm/min, and the stretching area was fixed at 10 mm.

## Supplementary information

Supplementary information.

## Data Availability

No datasets were generated or analysed during the current study.
