# Supplementary material for: Ag flake/silicone rubber composite with high stability and stretching speed insensitive resistance via conductive bridge formation
Source: Sci Rep. 2020 Mar 19;10:5036. doi: 10.1038/s41598-020-61752-2 (PMC7081184; doi:10.1038/s41598-020-61752-2)
Supplement: Supplementary file 1 — Supplementary information. [file 41598_2020_61752_MOESM1_ESM.docx]

**Ag flake/silicone rubber composite with high stability and stretching speed insensitive resistance via conductive bridge formation**

**In Seon Yoon^1,3^, Sun Hong Kim^2^, Youngsu Oh^1,3^, Byeong-Kwon Ju^1,*^, and Jae-Min Hong^3,4,*^**

^1^Display and Nanosystem Laboratory, Department of Electrical Engineering, Korea University, Seoul 02841, Republic of Korea

^2^Department of Electrical and Computer Engineering, Inter-University Semiconductor Research Centre, Seoul National University, Seoul 08826, Republic of Korea

^3^Photo-Electronic Hybrids Research Centre, Korea Institute of Science and Technology (KIST), Seoul 02792, Republic of Korea

^4^Institute of Advanced Composite Materials, Korea Institute of Science and Technology, Jeonbuk 55324, Republic of Korea

^*^Correspondence and requests for materials should be addressed to J.-M.H (email: jmhong@kist.re.kr) or B.-K.J. (email: bkju@korea.ac.kr)

**Supporting information**


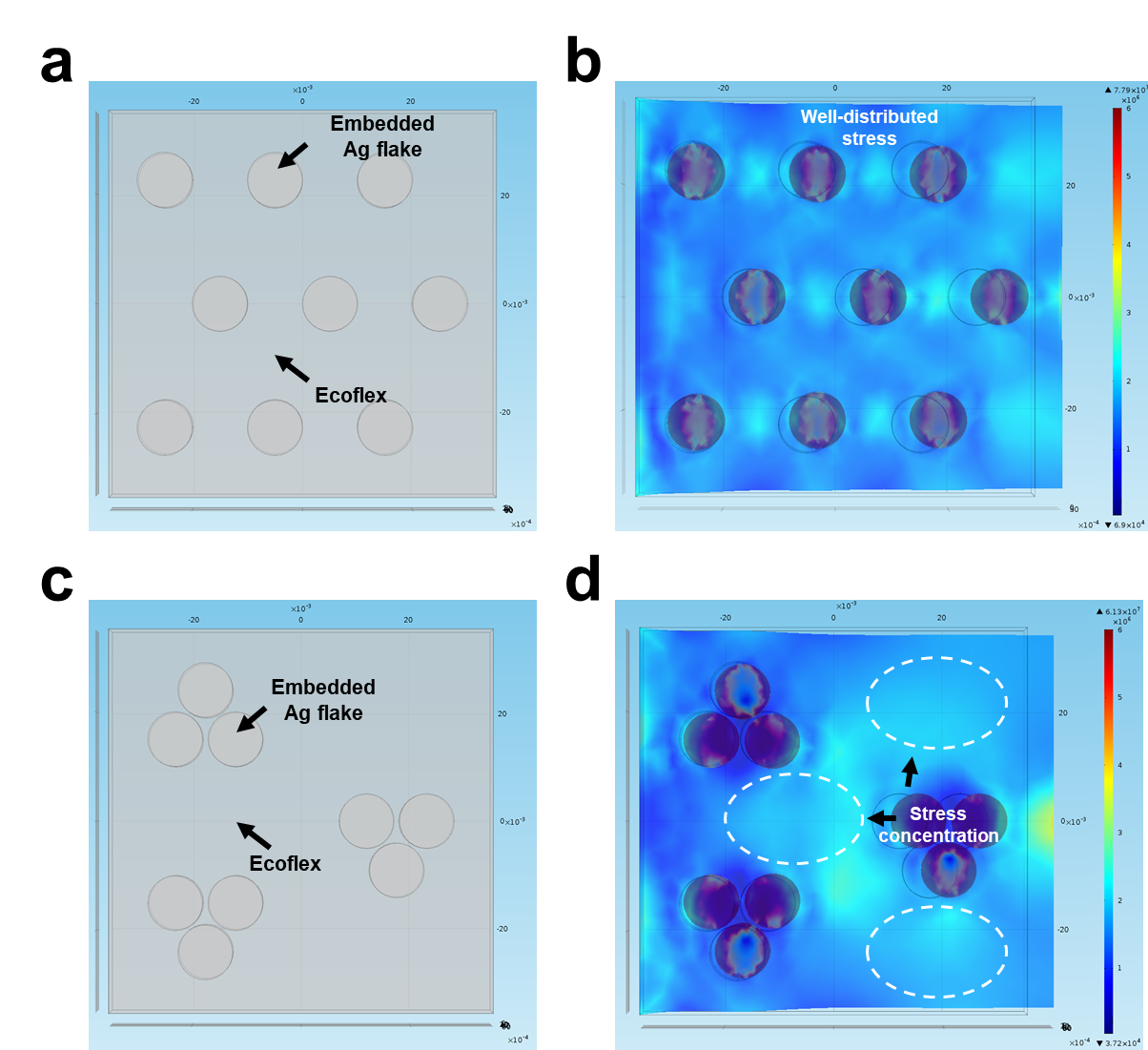


**Figure S1.** A schematic and simulation results for stress distribution. Schematic illustration of simulated structure of conductor with uniform dispersion (a) and simulation result (b). Schematic illustration of simulated structure of conductor with non-uniform dispersion (c) and simulation result (d). Stress-concentrated regions are found in non-uniformly dispersed composite which results in crack propagation. Stress distribution is simulated by the COMSOL multi-physics software.


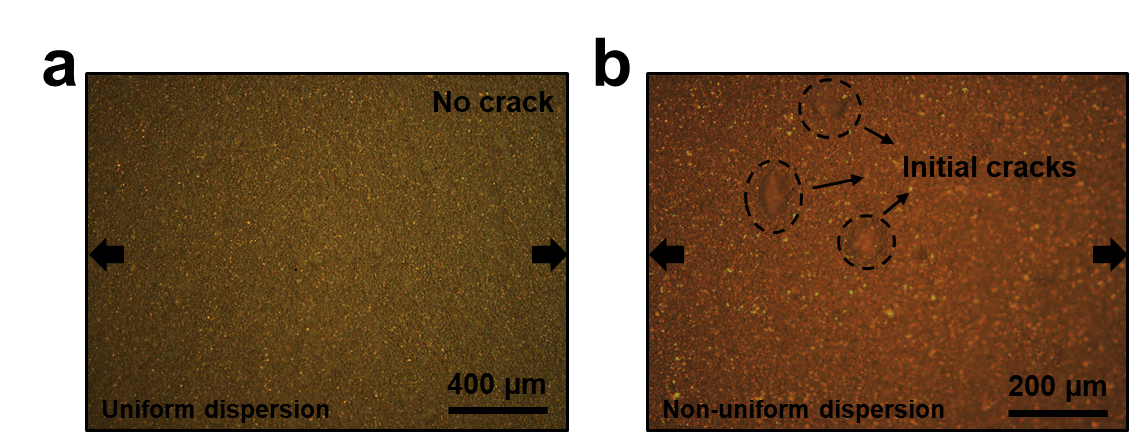


Figure S2. Optical microscopy images of stretched conductors after 5 cycles with uniform dispersion (a) and non-uniform dispersion (b) (100% strain).


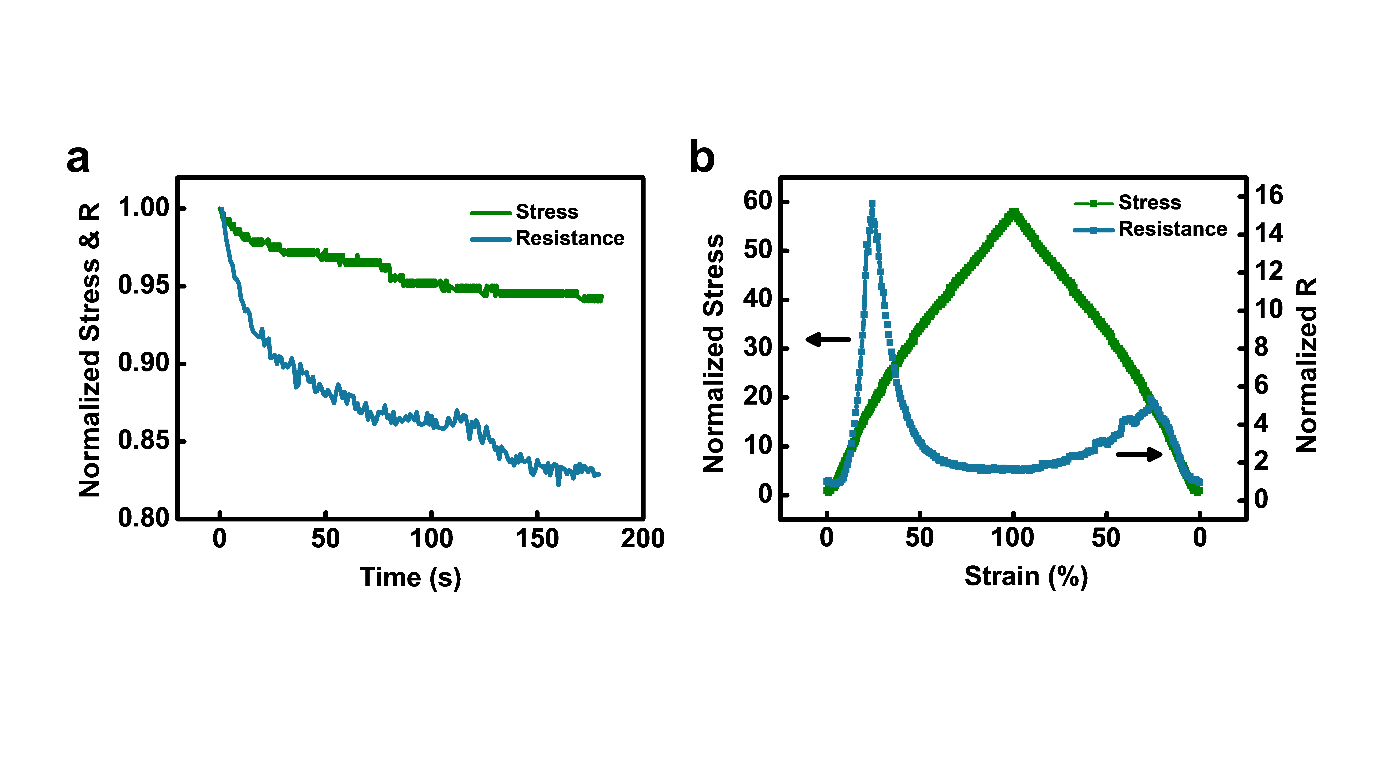


Figure S3. Normalized stress of the silicone rubber substrate and resistance variation of conductor as a function of (a) time under 300% strain and (b) cyclic strain (100%) after 9 cycles.


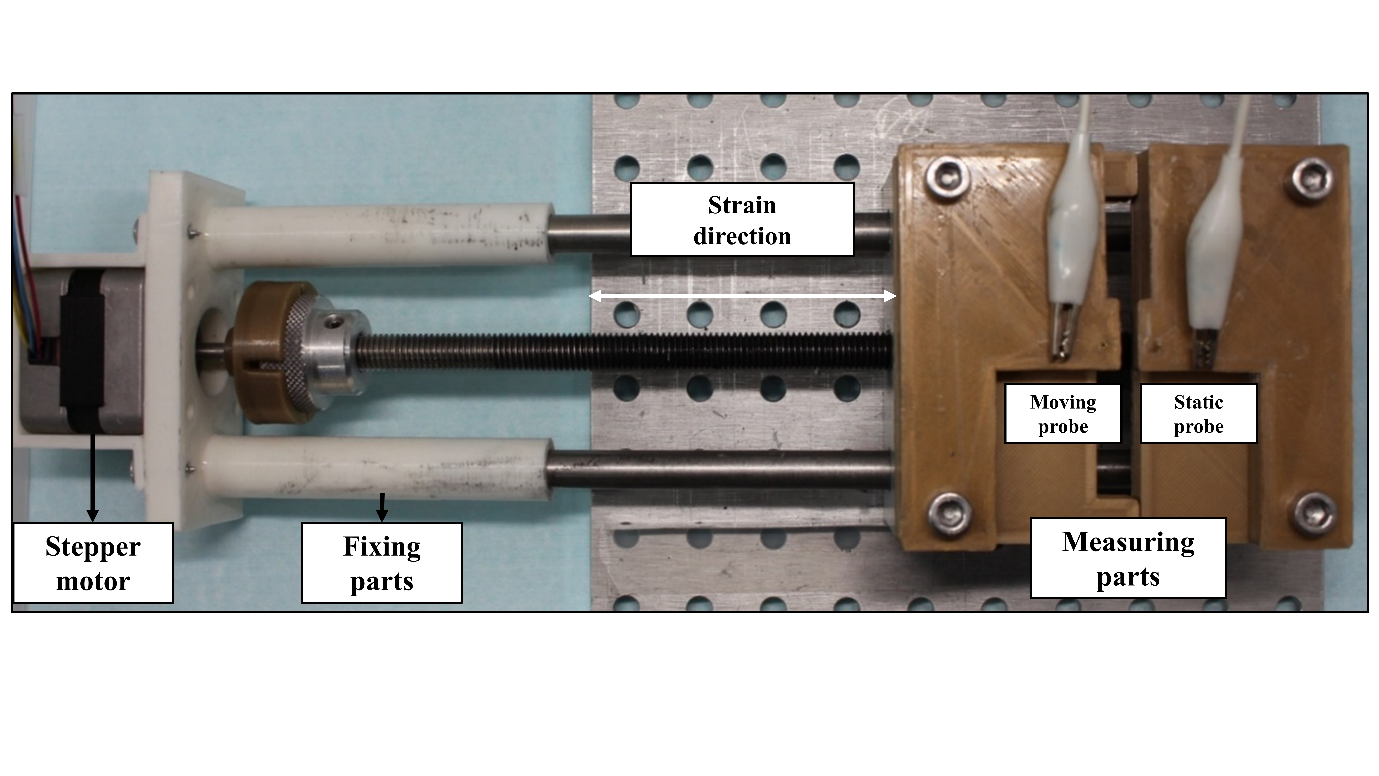


Figure S4. Photograph of the custom-made strain-testing system.

Figure S5. Magnitude of applied force to stretchable conductor up to 300% strain. The conductor is stretched at a speed of 5 mm/min.
